# Muscle ultrasound and its application to point-of-care ultrasonography: a narrative review

**DOI:** 10.1080/07853890.2022.2157871

**Published:** 2022-12-20

**Authors:** Masaaki Nagae, Hiroyuki Umegaki, Akito Yoshiko, Kosuke Fujita

**Affiliations:** a Department of Community Healthcare and Geriatrics, Nagoya University Graduate School of Medicine, Aichi, Japan; b Faculty of Liberal Arts and Sciences, Chukyo University, Aichi, Japan; c Department of Prevention and Care Science, Research Institute, National Center for Geriatrics and Gerontology, Aichi, Japan

**Keywords:** Point-of-care ultrasonography, muscle ultrasound, sarcopenia, clinical practice

## Abstract

Technological advances of hand-held ultrasound devices and educational programmes for their use, such as point-of-care ultrasonography (POCUS) training, have contributed to the increasing application of these devices in clinical practice. With the greater impact of frailty and sarcopenia in aging societies, attention is being focused on the use of ultrasound for skeletal muscle assessment. In this narrative review, we discuss how ultrasound can be applied to skeletal muscle assessment, especially that of the quadriceps muscle, in clinical practice. Muscle thickness by ultrasound has been shown to have good reliability and validity for the evaluation of muscle size, and echo intensity has been used to evaluate muscle quality. Muscle ultrasound has not only been useful to diagnose sarcopenia in various settings, but has also been validated to predict health-related outcomes such as death and functional disability. Recommended methods for muscle ultrasound was published recently, and the results of future studies are expected to be comparable. Although several challenging issues with muscle ultrasound remain, if it could be incorporated into educational programmes such as POCUS training, more clinicians may be able to use ultrasound for skeletal muscle assessment in the future.

**KEY MESSAGES**
- The evolution of hand-held ultrasound devices enables physicians to perform ultrasound at the bedside as part of regular medical examinations.
- Muscle ultrasound is considered an effective tool for evaluating muscle size and quality, and has been studied in various settings.
- More clinicians may be able to evaluate skeletal muscle assessment with the development of educational programmes on muscle ultrasound in the future.

## 1. Introduction

Ultrasound is widely used in clinical practice, contributing greatly to diagnosis and management of many conditions. While systematic ultrasound examinations have been conducted mainly by sonographers in an examination room, there is now considerable interest in having physicians perform ultrasound at the bedside, as part of regular medical examinations. This type of ultrasound examination is often called point-of-care ultrasonography (POCUS) and is characterized by its rapidity and limited focus in order to problem solve [1,2]. POCUS involves a somewhat simplified procedure compared with conventional ultrasound examinations and is designed to ensure a certain level of quality with the use of standardized decision criteria. POCUS training programmes have already been published by several organizations, including the American College of Emergency Physicians (ACEP) [3] and American Institute of Ultrasound in Medicine (AIUM) [4], and POCUS education has also been integrated into the curriculum for family medicine residents [5].

The development of POCUS is believed to be linked to the evolution of hand-held ultrasound devices that are smaller, cheaper and more portable. Studies using portable ultrasound have been spreading not only in the emergency room and intensive care unit (ICU) settings, but also in out-of-hospital situations in, for example, primary care and long-term care facilities (e.g. nursing homes). POCUS guides clinicians in conducting specific medical procedures as well as contributes to real-time clinical decision-making and management. Within the context of the ongoing COVID-19 pandemic, POCUS has been conducted to evaluate the severity of pneumonia, cardiac function, and deep vein thrombosis [6,7].

In recent years, POCUS has expanded into the fields of paediatrics, urology, otorhinolaryngology, and obstetrics and gynaecology. It is also receiving attention in the field of orthopaedics, such as for the assessment of rheumatoid arthritis, fracture, tendon injury and rotator cuff tear [8,9]. Moreover, ultrasound enables skeletal muscle to be evaluated. Actually, rehabilitative ultrasound imaging has been used to evaluate changes in skeletal muscle during therapeutic interventions [10]. Due to societal aging, the number of older people with physical frailty is increasing and the assessment of skeletal muscle is expected to be needed in various clinical situations.

As skeletal muscle assessment on ultrasound, trunk and neck muscles have been studied for assessing muscle size and quality [11–13]. However, muscle wasting is reported to be more prominent in the upper leg (thigh) muscle [14]. Thus, muscle ultrasound of the quadriceps muscle may be an effective way to sensitively reflect muscle atrophy and weakness in older adults. In this review, we discuss muscle ultrasound, especially that of the quadriceps muscle, and its application to POCUS for sarcopenia.

## 2. Methods

This narrative review was conducted to search the literature based on the PubMed and Cochrane databases. Full-text articles written in English were searched with no restriction on publication date and addressed muscle ultrasound in clinical practice. Search terms were a combination of the following MeSH terms (PubMed database): “Muscle, Skeletal[MeSH]”, “Quadriceps Muscle[Mesh]” , “ultrasonography[Mesh]”, “sarcopenia[Mesh]” and other terms “muscle mass”, “muscle size”, “muscle atrophy”, “muscle diameter”, “muscle volume”, “muscle thickness”, “muscle quality”, “echo intensity” and “echogenicity”. After reviewing the articles on muscle ultrasound, we categorized them into the following topics; assessment, measurement methods, clinical outcomes and problems. This narrative review was conducted according to the Scale for the Assessment of Narrative Review Articles (SANRA) guideline [15].

## 3. Discussion

### 3.1. Assessment of muscle size

Skeletal muscle is an essential focus when aiming to improve muscle strength and physical function. Traditionally, muscle ultrasound has been used in sports medicine or rehabilitation and has focused on the muscle mass changes related to training [16], bed rest [17], and aging [18].

Recently, with a number of basic and clinical studies focusing on sarcopenia, which is characterized by lower muscle mass, lower muscle strength and functional impairment [19], skeletal muscle assessment of older adults has been required in many situations. Muscle mass is commonly assessed by dual-energy X-ray absorptiometry (DXA) or bioelectrical impedance analysis (BIA) [20,21]. However, the former is expensive and involves radiation exposure, while the latter can be influenced by hydration status. In contrast, ultrasound has few of these limitations and can be performed at the bedside, making it more suitable for use with older adults. Muscle ultrasound has attracted attention for its ability to evaluate muscle morphology, and previous reviews have clarified the reliability and validity of the ultrasound-mediated quantification of muscle [22,23]. Furthermore, a recent systematic review reported moderate diagnostic value of muscle thickness (MT) for low muscle mass or sarcopenia (area under the curve = 0.76) and good validity of muscle mass calculated by ultrasound (*r* = 0.85–0.963) compared with DXA, with very high intra-rater and inter-rater reliabilities (ICC > 0.9) [24].

The MT of the quadriceps is the parameter most often evaluated by muscle ultrasound, and it has been shown to be a good predictor of muscle volume [25]. Muscle cross-sectional areas (CSAs) are also commonly evaluated.

### 3.2. Assessment of muscle quality

Although the assessment of muscle mass is considered most important in sarcopenia diagnosis, decreased muscle mass can only partially explain the loss of muscle strength, and the age-related decrease in muscle strength is much more rapid than that of muscle mass [26]. Indeed, the position statement of the Sarcopenia Definition and Outcomes Consortium (SDSC) states that muscle weakness, as determined by low grip strength, is a good predictor of adverse health-related outcomes, unlike DXA-derived muscle mass [27]. This implies that, beyond quantitative evaluations of muscle, qualitative changes are also important, such as micro- and macroscopic changes in muscle architecture and composition [20]. These qualitative changes are now referred to as muscle quality. There are no universally established methods for assessing muscle quality, although studies have used computed tomography, magnetic resonance imaging, and BIA to assess it by measuring fat infiltration into muscle or the phase angle [28].

For muscle ultrasound, echo intensity (EI) in skeletal muscle has been used to evaluate muscle quality. EI reflects the adipose and fibrous tissue within skeletal muscle. In other words, an increase in adipose and fibrous tissue within the muscle is visible as increased muscle brightness on ultrasound (hyperechogenicity). Indeed, the EI of the quadriceps femoris is known to be negatively correlated with quadriceps strength (knee extensor isometric strength) [29]. In addition to EI, other qualitative indicators for muscle are muscle fascicle length, pennation angle and muscle stiffness [30].

### 3.3. Methods of muscle ultrasound

Muscle atrophy progresses with age, with atrophy more predominant in the lower limbs than in the upper limbs. Numerous studies using muscle ultrasound have targeted the quadriceps muscles due to their relative ease of evaluation.

The position required at measurement is often supine, but some studies used the seated position. Linear-array probes (5–10 MHz) are commonly used, although convex probes are sometimes used. The rectus femoris (RF), RF + vastus intermedius and vastus lateralis are often evaluated, and the measurement points are typically the midpoint or two-thirds of the distance between the targeted muscles. Because the degree of pressure from the probe on the skin could easily affect the measurement value, a sufficient amount of water-soluble transmission gel is applied to the skin.

Therefore, muscle ultrasound is easily influenced by various conditions and is also operator-dependent. The measurement methods for muscle ultrasound were not standardized across many previous studies, but the SARCopenia through UltraSound (SARCUS) working group provided updated indications for the ultrasound protocol for muscle assessment in 2021 (Table 1) [30,31].

**Table 1.** Proposed anatomical landmarks of the quadriceps muscle [63].

	Proximal landmark	Distal landmark	Exact point	Remark
Rectus femoris	Greater trochanter	Proximal patella border	50%	Lying in neutral position
Vastus intermedius				
Vastus lateralis				

The results of any future studies performed following these recommendations would be comparable, which would improve understanding in this field.

When EI is being examined, the largest possible rectangular region of interest is determined manually, avoiding the visible surrounding fascia. EI can be measured using ImageJ software (National Institutes of Health, Bethesda, MD, USA) and expressed in arbitrary units 0 to 255, with a higher score indicating worse muscle quality [32].

**Figure 1.**
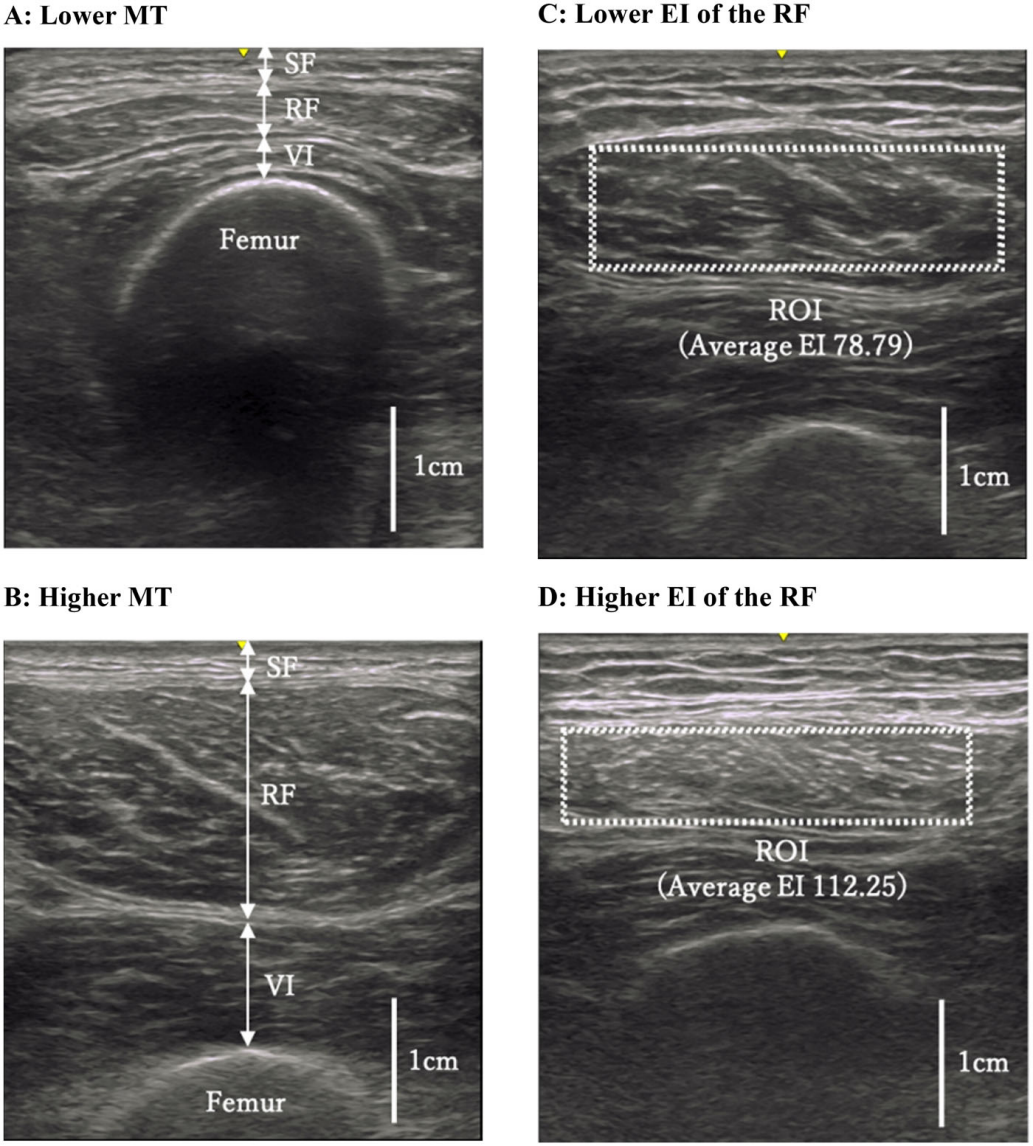
Ultrasound images of the quadriceps. (a,b) Thicknesses of the subcutaneous fat (SF), rectus femoris (RF) and vastus intermedius (VI) were measured at the position perpendicular to the femur in each image. (c,d) Dotted rectangle indicates maximum region of interest (ROI) in the RF. All images in Figure 1 were derived from our data, and have not been published elsewhere.

Figure 1 shows representative ultrasound images for MT (A, lower MT; B, higher MT) and EI (C, lower EI of the RF; D, higher EI of the RF).

### 3.4. Clinical outcomes assessed using muscle ultrasound

Muscle ultrasound is a validated tool for assessing muscle quantity in the diagnosis of sarcopenia [22] and has been studied in various settings [24]. Sarcopenia studies using muscle ultrasound have been conducted in not only older community-dwelling adults [33,34] and in outpatient settings [35,36], but also in individuals with comorbidities such as chronic obstructive pulmonary disease [37], heart failure [38], liver cirrhosis [39], haemodialysis [40], rheumatoid arthritis [41] and cancer [42]. Although muscle ultrasound has been described as an alternative indicator in the diagnosis of sarcopenia [20], the International Society of Physical and Rehabilitation Medicine (ISPRM) recently proposed a new diagnostic algorithm that uses muscle ultrasound rather than conventional methods for muscle mass assessment [43]. In this algorithm, the sonographic thigh adjustment ratio, which is calculated as the anterior thigh MT divided by body mass index, is recommended for evaluating loss of muscle mass (<1.0 for females and <1.4 for males) [44].

Moreover, attention has recently turned to whether muscle ultrasound can predict health-related outcomes in clinical practice. A systematic review reported that MT, CSA and EI were significantly associated with physical function, length of hospital stay, readmission and survival [45]. Many studies have been conducted in the ICU, with some examining the association between muscle changes and ICU-acquired weakness (ICU-AW) or mortality from muscle ultrasound results over time. One study showed that decreased MT or CSA in the lower extremities at 10 days (15% and 12%, respectively) had good diagnostic accuracy for ICU-AW [46] while another study showed that an elevated EI of the RF at 7 days predicted ICU-AW at hospital discharge [47]. In terms of mortality, a recent study found that a decrease in quadriceps MT at 7 days was an independent predictor of 60-day mortality [48], whereas another study of patients with COVID-19 found that reduced CSA and EI of the RF at 7 days were also associated with death during ICU stays [49].

Some studies that explored the association between muscle ultrasound and adverse outcomes have been conducted outside the ICU setting. For example, Akazawa et al. [50–52] investigated the association between EI and activities of daily living in patients admitted to a subacute and convalescent rehabilitation ward. Moreover, Nagae et al. [53,54] reported the association between EI and hospital-associated complications and also the association between MT and hospital-associated disability in acute hospitalized older adults. Yamada et al. [55] combined indicators of MT and EI and reported their association with the occurrence of falls in community-dwelling older adults.

### 3.5. Challenges and limitations of muscle ultrasound

As mentioned above, muscle ultrasound is often useful for sarcopenia diagnosis and outcome prediction. Therefore, more studies will undoubtedly be conducted using muscle ultrasound into the future. However, muscle ultrasound is associated with several problems. First, cut-off values for diagnosing low muscle mass or sarcopenia by ultrasound have not been determined. Anthropometric and sociodemographic characteristics could easily affect the values of muscle mass. Some studies calculated the cut-off values of MT and CSA [24], but large multicenter studies are required to confirm these. Similarly, there are no clear cut-off values for EI. Second, examiners require a certain level of experience, and inter-rater reliability has sometimes been a concern, although large muscles such as the quadriceps are quite clearly detectable. Third, particularly in the acute phase, muscle quantity and quality values could be changeable. MT is considered to temporarily increase with systemic inflammation and elevated vascular permeability [56], and EI is also affected by hydration, intra-/extracellular fluid valance, and glycogen levels as well as muscle damage [32]. Thus, MT and EI may not always accurately reflect actual muscle mass and muscle quality. Fourth, as a property of ultrasound, EI, especially in deep muscle, can lead to the calculation of inaccurate values because EI attenuates with depth. Although Young et al. [57] have developed a formula to correct EI by subcutaneous fat thickness, it is unclear whether EI or corrected EI more accurately reflects muscle quality in clinical practice. Moreover, the EI value could vary among ultrasound instruments or setting conditions and it may be challenging to compare results among studies.

### 3.6. Future direction

While several challenging problems remain, establishing an educational programme for muscle ultrasound may be a turning point. In fact, even beginners at muscle ultrasound can detect MT and EI on quadriceps images and show good intra-examiner reliability after only a short period of instruction [58]. Using a phantom model for the RF was also shown to improve the accuracy of muscle ultrasound measurements conducted by inexperienced healthcare providers [59]. These findings suggest that education programmes or use of training models can expand the use of muscle ultrasound in clinical settings.

There are also emerging reports concerning the use of portable ultrasound for the assessment of muscle. Madden et al. examined MT of the vastus medialis using a Vscan™ with Dual Probe hand-held ultrasound (GE Healthcare, IL, USA) and showed strong correlations with lean body mass and muscle strength [60] and a weak negative correlation with frailty [61]. Canales et al. [62] examined the quadriceps depth in preoperative patients using a Vivid S6 ultrasound machine (GE Healthcare) and demonstrated good ability to identify frailty and associations with the adverse outcomes of unplanned postoperative skilled nursing facility discharge disposition and delirium. Other hand-held devices with a high resolution are currently available, and it is becoming possible to store and edit images wirelessly. Numerous studies will likely use these new ultrasound devices to not only measure MT, but also EI or other parameters in a wide range of settings, including in community-dwelling adults and nursing home residents.

Some educational programmes for POCUS are already available in the musculoskeletal field [8,9], but these programmes mainly involve shoulder, knee, spine and limb joint evaluations and focus on rapid diagnosis and intervention. Older adults often have multimorbidity, which is closely linked to new-onset sarcopenia [63]. In clinical practice with older adults, approaches to those problems that might impair independence and quality of life are considered to be highly important, as well as the early detection and management of acute diseases. In other words, muscle assessment in regular medical care for older adults may be worthwhile. If muscle ultrasound that evaluates muscle mass and quality could be incorporated as one of the POCUS-related measurements in the musculoskeletal region, more clinicians will perform muscle assessment. However, clear diagnostic and decision criteria for muscle ultrasound should be established, and more studies will also be needed to clarify the correlations between conventional ultrasound and portable ultrasound instruments.

## 4. Conclusions

Due to the importance of frailty and sarcopenia in aging societies, evaluating muscle size and quality is required in various settings. Ultrasound is one of the most reliable modalities for evaluating skeletal muscle assessment and has the advantage of simplicity and reproducibility in clinical practice. Technological innovations in portable ultrasound have also contributed to muscle assessments being conducted. Currently, several challenging issues with muscle ultrasound remain. However, educational approaches, such as those performed for POCUS, may provide an opportunity to expand the future application of muscle ultrasound.

## Data Availability

Data sharing is not applicable to this article because no new data were created or analysed.

## References

[1] Moore CL, Copel JA. Point-of-care ultrasonography. N Engl J Med. 2011;364(8):749–757. 21345104 10.1056/NEJMra0909487

[2] Díaz-Gómez JL, Mayo PH, Koenig SJ. Point-of-care ultrasonography. N Engl J Med. 2021;385(17):1593–1602. 34670045 10.1056/NEJMra1916062

[3] Statement P. Ultrasound guidelines: emergency, point-of-care and clinical ultrasound guidelines in medicine. Ann Emerg Med. 2017;69:e27–e54. 28442101 10.1016/j.annemergmed.2016.08.457

[4] Emergency P, Guidelines U. AIUM practice parameter for the performance of point-of-care ultrasound examinations. J Ultrasound Med. 2019;38:833–849. 30895665 10.1002/jum.14972

[5] American Academy of Family Physicians. Recommended curriculum guidelines for family medicine residents Point of care ultrasound. https://www.aafp.org/dam/AAFP/documents/medical_education_residency/program_directors/Reprint290D_POCUS.pdf. Accessed August 30, 2022.

[6] Schrift D, Barron K, Arya R, et al. The use of POCUS to manage ICU patients with COVID-19. J Ultrasound Med. 2021;40(9):1749–1761. 33174650 10.1002/jum.15566

[7] Johri AM, Galen B, Kirkpatrick JN, et al. ASE statement on point-of-care ultrasound during the 2019 novel coronavirus pandemic. J Am Soc Echocardiogr. 2020;33(6):670–673. 32503704 10.1016/j.echo.2020.04.017 PMC7158805

[8] Chen KC, Lin ACM, Chong CF, et al. An overview of point-of-care ultrasound for soft tissue and musculoskeletal applications in the emergency department. J Intensive Care. 2016;4(1):11. 10.1186/s40560-016-0173-0 PMC4983782 27529031

[9] Arnold MJ, Jonas CE, Carter RE. Point-of-care ultrasonography. Am Fam Physician. 2020;101(5):275–285. 32109031

[10] Valera-Calero JA, Fernández-de-Las-Peñas C, Varol U, et al. Ultrasound imaging as a visual biofeedback tool in rehabilitation: an updated systematic review. IJERPH. 2021;18(14):7554. 34300002 10.3390/ijerph18147554 PMC8305734

[11] Plaza-Manzano G, Navarro-Santana MJ, Valera-Calero JA, et al. Reliability of lumbar multifidus ultrasound assessment during the active straight leg raise test. Eur J Clin Invest. 2022;52(5):e13728. 34882303 10.1111/eci.13728

[12] Valera-Calero JA, Arias-Buría JL, Fernández-de-Las-Peñas C, et al. Echo-intensity and fatty infiltration ultrasound imaging measurement of cervical multifidus and short rotators in healthy people: a reliability study. Musculoskelet Sci Pract. 2021;53:102335. 33531271 10.1016/j.msksp.2021.102335

[13] Valera-Calero JA, Gallego-Sendarrubias GM, Fernández-de-Las-Peñas C, et al. Panoramic ultrasound examination of posterior neck extensors in healthy subjects: intra-Examiner reliability study. Diagnostics. 2020;10(10):740. 32987741 10.3390/diagnostics10100740 PMC7598691

[14] Abe T, Loenneke JP, Thiebaud RS, et al. Age-related site-specific muscle wasting of upper and lower extremities and trunk in Japanese men and women. Age. 2014;36(2):813–821. 24243442 10.1007/s11357-013-9600-5 PMC4039273

[15] Baethge C, Goldbeck-Wood S, Mertens S. SANRA-a scale for the quality assessment of narrative review articles. Res Integr Peer Rev. 2019;4:5. 30962953 10.1186/s41073-019-0064-8 PMC6434870

[16] Ikai M, Fukunaga T. A study on training effect on strength per unit cross-sectional area of muscle by means of ultrasonic measurement. Int Z Angew Physiol. 1970;28(3):173–180. 5425330 10.1007/BF00696025

[17] Kawakami Y, Muraoka Y, Kubo K, et al. Changes in muscle size and architecture following 20 days of bed rest. J Gravit Physiol. 2000;7(3):53–59. 12124185

[18] Ishida Y, Kanehisa H, Carroll JF, et al. Distribution of subcutaneous fat and muscle thicknesses in young and middle-aged women. Am J Hum Biol. 1997;9(2):247–255. 28561521 10.1002/(SICI)1520-6300(1997)9:2<247::AID-AJHB11>3.0.CO;2-M

[19] Cruz-Jentoft AJ, Sayer AA. Sarcopenia. The Lancet. 2019;393(10191):2636–2646. 10.1016/S0140-6736(19)31138-9 31171417

[20] Cruz-Jentoft AJ, Bahat G, Bauer J, et al, Writing Group for the European Working Group on Sarcopenia in Older People 2 (EWGSOP2), and the Extended Group for EWGSOP2. Sarcopenia: revised European consensus on definition and diagnosis. Age Ageing. 2019;48(1):16–31. 30312372 10.1093/ageing/afy169 PMC6322506

[21] Chen LK, Woo J, Assantachai P, et al. Asian Working Group for Sarcopenia: 2019 Consensus Update on sarcopenia diagnosis and treatment. J Am Med Dir Assoc. 2020;21(3):300–307.e302. 32033882 10.1016/j.jamda.2019.12.012

[22] Nijholt W, Scafoglieri A, Jager-Wittenaar H, et al. The reliability and validity of ultrasound to quantify muscles in older adults: a systematic review. J Cachexia Sarcopenia Muscle. 2017;8(5):702–712. 28703496 10.1002/jcsm.12210 PMC5659048

[23] Ticinesi A, Meschi T, Narici MV, et al. Muscle ultrasound and sarcopenia in older individuals: a clinical perspective. J Am Med Dir Assoc. 2017;18(4):290–300. 28202349 10.1016/j.jamda.2016.11.013

[24] Zhao R, Li X, Jiang Y, et al. Evaluation of appendicular muscle mass in sarcopenia in older adults using ultrasonography: a systematic review. Gerontology. 2022;68(10):1174–1198. 35878591 10.1159/000525758 PMC9677864

[25] Miyatani M, Kanehisa H, Ito M, et al. The accuracy of volume estimates using ultrasound muscle thickness measurements in different muscle groups. Eur J Appl Physiol. 2004;91(2–3):264–272. 14569399 10.1007/s00421-003-0974-4

[26] Goodpaster BH, Park SW, Harris TB, et al. The loss of skeletal muscle strength, mass, and quality in older adults: the health, aging and body composition study. J Gerontol A Biol Sci Med Sci. 2006;61(10):1059–1064. 17077199 10.1093/gerona/61.10.1059

[27] Bhasin S, Travison TG, Manini TM, et al. Sarcopenia definition: the position statements of the sarcopenia definition and outcomes consortium. J Am Geriatr Soc. 2020;68(7):1410–1418. 32150289 10.1111/jgs.16372 PMC12132920

[28] Akamatsu Y, Kusakabe T, Arai H, et al. Phase angle from bioelectrical impedance analysis is a useful indicator of muscle quality. J Cachexia Sarcopenia Muscle. 2022;13(1):180–189. 34845859 10.1002/jcsm.12860 PMC8818694

[29] Fukumoto Y, Ikezoe T, Yamada Y, et al. Skeletal muscle quality assessed from echo intensity is associated with muscle strength of middle-aged and elderly persons. Eur J Appl Physiol. 2012;112(4):1519–1525. 21847576 10.1007/s00421-011-2099-5

[30] Perkisas S, Bastijns S, Baudry S, et al. Application of ultrasound for muscle assessment in sarcopenia: 2020 SARCUS update. Eur Geriatr Med. 2021;12(1):45–59. 33387359 10.1007/s41999-020-00433-9

[31] Perkisas S, Baudry S, Bauer J, et al. Application of ultrasound for muscle assessment in sarcopenia: towards standardized measurements. Eur Geriatr Med. 2018;9(6):739–757. 34674473 10.1007/s41999-018-0104-9

[32] Stock MS, Thompson BJ. Echo intensity as an indicator of skeletal muscle quality: applications, methodology, and future directions. Eur J Appl Physiol. 2021;121(2):369–380. 33221942 10.1007/s00421-020-04556-6

[33] Yamada M, Kimura Y, Ishiyama D, et al. Differential characteristics of skeletal muscle in community-dwelling older adults. J Am Med Dir Assoc. 2017;18:807.e809–807.e816. 10.1016/j.jamda.2017.05.011 28676289

[34] Fukumoto Y, Ikezoe T, Taniguchi M, et al. Cut-off values for lower limb muscle thickness to detect low muscle mass for sarcopenia in older adults. Clin Interv Aging. 2021;16:1215–1222. 34211270 10.2147/CIA.S304972 PMC8241812

[35] Ozturk Y, Koca M, Burkuk S, et al. The role of muscle ultrasound to predict sarcopenia. Nutrition. 2022;101:111692–111692. 35660496 10.1016/j.nut.2022.111692

[36] Rustani K, Kundisova L, Capecchi PL, et al. Ultrasound measurement of rectus femoris muscle thickness as a quick screening test for sarcopenia assessment. Arch Gerontol Geriatr. 2019;83:151–154. 31029040 10.1016/j.archger.2019.03.021

[37] Deng M, Zhou X, Li Y, et al. Ultrasonic elastography of the rectus femoris, a potential tool to predict sarcopenia in patients with chronic obstructive pulmonary disease. Front Physiol. 2021;12:783421. 35069243 10.3389/fphys.2021.783421 PMC8766419

[38] Sato Y, Shiraishi H, Nakanishi N, et al. Clinical significance of rectus femoris diameter in heart failure patients. Heart Vessels. 2020;35(5):672–680. 31701229 10.1007/s00380-019-01534-7

[39] Dhariwal S, Roy A, Taneja S, et al. Assessment of Sarcopenia Using Muscle Ultrasound in Patients with Cirrhosis and Sarcopenic Obesity (AMUSE STUDY). J Clin Gastroenterol. Published online 2022 Aug 9. 10.1097/MCG.0000000000001745 35943413

[40] Matsuzawa R, Yamamoto S, Suzuki Y, et al. The clinical applicability of ultrasound technique for diagnosis of sarcopenia in hemodialysis patients. Clin Nutr. 2021;40(3):1161–1167. 32798065 10.1016/j.clnu.2020.07.025

[41] Yoshida T, Kumon Y, Takamatsu N, et al. Ultrasound assessment of sarcopenia in patients with rheumatoid arthritis. Mod Rheumatol. 2022;32(4):728–735. 34897497 10.1093/mr/roab049

[42] Gomes TLN, Borges TC, Pichard C, et al. Correlation between SARC-F score and ultrasound-measured thigh muscle thickness in older hospitalized cancer patients. J Nutr Health Aging. 2020;24(10):1128–1130.33244572 10.1007/s12603-020-1524-z

[43] Kara M, Kaymak B, Frontera WR, et al. Diagnosing sarcopenia: functional perspectives and a new algorithm from ISarcoPRM. J Rehabil Med. 2021;53(6):jrm00209. 34121127 10.2340/16501977-2851 PMC8814891

[44] Kara M, Kaymak B, Ata AM, et al. STAR – Sonographic Thigh Adjustment Ratio: a golden formula for the diagnosis of sarcopenia. Am J Phys Med Rehabil. 2020;99(10):902–908. 32941253 10.1097/PHM.0000000000001439

[45] Casey P, Alasmar M, McLaughlin J, et al. The current use of ultrasound to measure skeletal muscle and its ability to predict clinical outcomes: a systematic review. J Cachexia Sarcopenia Muscle. 2022;13(5):2298–2309. 35851996 10.1002/jcsm.13041 PMC9530572

[46] Zhang W, Wu J, Gu Q, et al. Changes in muscle ultrasound for the diagnosis of intensive care unit acquired weakness in critically ill patients. Sci Rep. 2021;11(1):18280. 34521934 10.1038/s41598-021-97680-y PMC8440559

[47] Mayer KP, Thompson Bastin ML, Montgomery-Yates AA, et al. Acute skeletal muscle wasting and dysfunction predict physical disability at hospital discharge in patients with critical illness. Crit Care. 2020;24(1):637–637. 33148301 10.1186/s13054-020-03355-x PMC7640401

[48] Lee ZY, Ong SP, Ng CC, et al. Association between ultrasound quadriceps muscle status with premorbid functional status and 60-day mortality in mechanically ventilated critically ill patient: a single-center prospective observational study. Clin Nutr. 2021;40(3):1338–1347. 32919818 10.1016/j.clnu.2020.08.022

[49] Umbrello M, Guglielmetti L, Formenti P, et al. Qualitative and quantitative muscle ultrasound changes in covid-19 related ards patients. Nutrition. 2021;91-92:111449–111449. 34583135 10.1016/j.nut.2021.111449 PMC8364677

[50] Akazawa N, Kishi M, Hino T, et al. Increased intramuscular adipose tissue of the quadriceps is more strongly related to declines in ADL than is loss of muscle mass in older inpatients. Clin Nutr. 2021;40(3):1381–1387. 32917418 10.1016/j.clnu.2020.08.029

[51] Akazawa N, Kishi M, Hino T, et al. Intramuscular adipose tissue in the quadriceps is more strongly related to recovery of activities of daily living than muscle mass in older inpatients. J Cachexia Sarcopenia Muscle. 2021;12(4):891–899. 33998169 10.1002/jcsm.12713 PMC8350216

[52] Akazawa N, Kishi M, Hino T, et al. Longitudinal relationship between intramuscular adipose tissue of the quadriceps and activities of daily living in older inpatients. J Cachexia Sarcopenia Muscle. 2021;12(6):2231–2237. 34704384 10.1002/jcsm.12842 PMC8718049

[53] Nagae M, Umegaki H, Yoshiko A, et al. Echo intensity is more useful in predicting hospital-associated complications than conventional sarcopenia-related parameters in acute hospitalized older patients. Exp Gerontol. 2021;150:111397–111397. 33965558 10.1016/j.exger.2021.111397

[54] Nagae M, Umegaki H, Yoshiko A, et al. Muscle evaluation and hospital-associated disability in acute hospitalized older adults. J Nutr Health Aging. 2022;26(7):681–687. 35842758 10.1007/s12603-022-1814-8 PMC9194346

[55] Yamada M, Kimura Y, Ishiyama D, et al. Combined effect of lower muscle quality and quantity on incident falls and fall-related fractures in community-dwelling older adults: a 3-year follow-up study. Bone. 2022;162:116474–116474. 35752409 10.1016/j.bone.2022.116474

[56] Welch C, Greig CA, Hassan-Smith ZK, et al. A pilot observational study measuring acute sarcopenia in older colorectal surgery patients. BMC Res Notes. 2019;12(1):24. 30642375 10.1186/s13104-019-4049-y PMC6332645

[57] Young HJ, Jenkins NT, Zhao Q, et al. Measurement of intramuscular fat by muscle echo intensity. Muscle Nerve. 2015;52(6):963–971. 25787260 10.1002/mus.24656 PMC4575231

[58] Abiko T, Ohmae K, Murata S, et al. Reliability of muscle thickness and echo intensity measurements of the quadriceps: a novice examiner. J Bodyw Mov Ther. 2022;31:164–168. 35710216 10.1016/j.jbmt.2022.03.004

[59] Nakanishi N, Inoue S, Tsutsumi R, et al. Rectus femoris mimicking ultrasound phantom for muscle mass assessment: design, research, and training application. JCM. 2021;10(12):2721–2721. 34202957 10.3390/jcm10122721 PMC8235438

[60] Madden KM, Feldman B, Arishenkoff S, et al. A rapid point-of-care ultrasound marker for muscle mass and muscle strength in older adults. Age Ageing. 2021;50(2):505–510. 32909032 10.1093/ageing/afaa163 PMC7936023

[61] Madden KM, Feldman B, Arishenkoff S, et al. Point-of-care ultrasound measures of muscle and frailty measures. Eur Geriatr Med. 2021;12(1):161–166. 32960448 10.1007/s41999-020-00401-3

[62] Canales C, Mazor E, Coy H, et al. Preoperative point-of-care ultrasound to identify frailty and predict postoperative outcomes: a diagnostic accuracy study. Anesthesiology. 2022;136(2):268–278. 34851395 10.1097/ALN.0000000000004064 PMC9843825

[63] Veronese N, Smith L, Cereda E, et al. Multimorbidity increases the risk for sarcopenia onset: longitudinal analyses from the English longitudinal study of ageing. Exp Gerontol. 2021;156:111624–111624. 34767942 10.1016/j.exger.2021.111624

